# Determinants of Cancer Screening in India: An Epidemiological Overview

**DOI:** 10.7759/cureus.102339

**Published:** 2026-01-26

**Authors:** Vijna, Dharmendra Kumar Dubey, Pramod Mishra

**Affiliations:** 1 Department of Community Medicine, Baba Kinaram Autonomous State Medical College, Chandauli, IND; 2 Department of Preventive Medicine, Padmini Care Hospital, Dhaneswar Rath Institute of Engineering and Management Studies (DRIEMS) University, Tangi, IND

**Keywords:** breast cancer, cancer screening, cervical cancer, india, nfhs

## Abstract

Background: Noncommunicable diseases (NCDs) have emerged as a major global health burden. Among these, cancer is the second leading cause of mortality worldwide and accounts for a substantial proportion of deaths in India. This study aimed to evaluate state-specific disparities in breast, cervical, and oral cancer screening coverage and to identify determinants of cancer screening patterns.

Methods and materials: Data relevant to the study objectives were obtained from the fifth National Family Health Survey, which follows a cross-sectional study design. For analysis, only women aged 15-49 years were included. The primary outcomes of interest were self-reported screening behaviors for cervical, breast, and oral cancers. Responses were dichotomized as “yes” (screened) or “no” (not screened). Three separate models, each focusing on a distinct set of independent variables, were used to assess determinants of cancer screening behavior. Associations between outcome and predictor variables were examined using chi-square tests. Logistic regression models were used to estimate the likelihood of undergoing various cancer screening tests in relation to selected independent variables.

Results: This study revealed alarmingly low cancer screening rates in India. At the national level, only 1.2% (n = 2,642) of women reported screening for cervical cancer, 0.6% (n = 1,421) for oral cancer, and 0.5% (n = 1,230) for breast cancer.

Conclusions: Exposure to health-related messages through television and radio was positively associated with cancer screening uptake. Higher body mass index and the spouse's educational status were also positive predictors of screening participation. In contrast, high parity, current breastfeeding status, and rural residence were associated with lower screening uptake. The history of terminated pregnancy, employment status, and exposure to print media were not significant influencers. To address sociodemographic disparities in screening coverage, targeted interventions, such as mobile screening units in underserved rural areas, strengthened primary healthcare services, and community-based outreach through frontline health workers, should be prioritized. Additionally, culturally tailored awareness campaigns and strengthened referral linkages may further enhance screening uptake among disadvantaged populations.

## Introduction

Noncommunicable diseases (NCDs) have emerged as a pervasive global health burden. In 2021, an estimated 43 million deaths were attributed to NCDs, of which 18 million occurred prematurely before the age of 70. These deaths accounted for over 75% of all non-pandemic-related mortality worldwide. Alarmingly, 82% of these deaths occurred in low- and middle-income countries [1].

In 2022, cancer was the second leading contributor to global mortality among NCDs. Nearly half of all individuals diagnosed with cancer succumbed to the disease in the same year, with 9.7 million deaths reported among 20 million new cases worldwide [2]. Asia, home to the largest share of the global population, bore a disproportionate burden, accounting for 49.2% of new cancer cases and 56.1% of cancer-related deaths globally.

Breast cancer emerged as the most frequently diagnosed malignancy, accounting for 2.2 million new cases (11.3% of all cancer diagnoses) and 7.0% of cancer-related deaths worldwide. Cervical cancer, another major public health concern, accounted for 6.0 million new cases (3.0%) and 3.4 million deaths (3.5%). Oral cancer contributed 3.8 million new cases (1.9%) and 1.8 million deaths (1.8%) globally [3,4].

According to the World Health Organization NCD Country Profile for India, the national burden of NCDs remains substantial. NCDs account for approximately 63% of all deaths in India, with cancers contributing nearly 9% of total NCD-related mortality [5,6]. In 2022, India recorded approximately 917,000 cancer-related deaths among 1.41 million individuals (0.1% of the population) diagnosed with cancer. Among women, breast cancer emerged as the leading cause of cancer-related mortality, with 190,000 new cases (13.6%) and 98,337 deaths. Cervical cancer followed, with 130,000 cases (9.0%) and 79,906 deaths (8.7%). Predominantly affecting men, oral cancer accounted for 143,000 new cases (10.2%) and 79,979 deaths (8.7%), ranking second overall among all cancers in the same year [2].

Projections from the Indian Council of Medical Research-National Cancer Registry Programme indicate a concerning rise in India’s cancer burden, from 1.4 million cases in 2022 to an estimated 1.57 million cases by 2025 [7]. Despite the presence of public health schemes, approximately 75% of cancer-related healthcare expenditures are incurred out of pocket, with cancer care costs estimated to be nearly six times higher than those for infectious diseases [8-12]. The escalating incidence of cancer thus imposes a substantial financial burden on affected households [13]. Notably, cancer-related hospitalizations carry a 160% higher risk of catastrophic health expenditure compared with communicable diseases and incur nearly twice the costs associated with injuries or cardiovascular conditions [14].

Screening for oral, breast, and cervical cancers has been shown to substantially improve five-year survival rates, increasing to nearly 60% when cancers are detected at an early stage but declining to below 15% when diagnosed at advanced stages [15]. Accordingly, screening programs aim to detect early, asymptomatic, and precancerous lesions [16]. Evidence suggests that human papillomavirus vaccination and screening can prevent up to 95% of cervical cancer cases and reduce mortality by as much as 80% [17]. In Kerala, India, periodic mass and targeted oral visual screening in high-risk populations significantly reduced oral cancer mortality [18]. To scale up such preventive efforts, the Government of India introduced population-based screening under the National Health Mission through the National Programme for Prevention and Control of Noncommunicable Diseases (NP-NCD), implemented via Ayushman Bharat Health and Wellness Centres [19].

To assess the gap between policy intent and field-level implementation of population-based cancer screening, the National Family Health Survey-5 (NFHS-5) offers a valuable platform for evaluating coverage and effectiveness [20]. Given India’s substantial contribution to the global cancer burden, these initiatives have the potential to significantly reduce cancer-related morbidity and mortality nationwide. However, a paucity of nationally representative analyses disaggregated by cancer type and state-level variation remains a critical research gap. Although NFHS-5 provides macro-level estimates, evidence on micro-level sociodemographic determinants influencing screening uptake is limited.

To address this gap, a cross-sectional analytical study using NFHS-5 data was conducted. The analysis was restricted to women aged 15-49 years and aimed to assess state-specific disparities in breast, cervical, and oral cancer screening coverage, as well as to identify key determinants associated with cancer screening patterns.

## Materials and methods

The NFHS-5, conducted between 2019 and 2021, introduced questions on cancer screening for the first time. This study aimed to assess the prevalence of screening for cervical, breast, and oral cancers among 225,565 women respondents aged 15-49 years. The analysis included women with complete information on cancer screening outcomes and relevant covariates; respondents with missing, incomplete, or “don’t know” responses for key outcome or explanatory variables were excluded. NFHS-5 employed a multistage sampling design, selecting households from both rural and urban areas across all 707 districts in India. Data were collected using computer-assisted personal interviewing with standardized questionnaires adopted from the NFHS-5 biomarker module, which is publicly available and open access, allowing efficient data collection and real-time data entry [21].

Chi-square tests were used to examine associations between outcome and predictor variables. Binary logistic regression models were employed to assess the relationship between selected independent variables and the likelihood of undergoing different cancer screening tests. Odds ratios (ORs) with 95% confidence intervals (CIs) were calculated to determine the strength and direction of associations. The dependent variable was a binary indicator representing whether a woman had ever undergone screening for cervical, breast, or oral cancer, based on the survey question, “Have you ever undergone a screening test for cervical, breast, or oral cancer?” Responses were coded as yes = 1 (screened) and no = 0 (not screened).

A domain-wise modeling approach was adopted, with three separate multivariable logistic regression models constructed to evaluate the influence of distinct predictor groups.

Model 1 (sociodemographic determinants) assessed screening patterns based on sociodemographic characteristics. The independent variables included were age ($x_1$), place of residence ($x_2$), education ($x_3$), caste ($x_4$), religion ($x_5$), and wealth index ($x_6$).

​​​​​​Model 2 (reproductive determinants) evaluated screening patterns based on reproductive factors, including birth order ($x_1$), history of terminated pregnancy ($x_2$), current breastfeeding status ($x_3$), current marital status ($x_4$), and current co-residence with husband/partner ($x_5$).

Model 3 (ancillary determinants) examined screening patterns based on ancillary factors, including working status ($x_1$), body mass index (BMI) ($x_2$), husband/partner’s education ($x_3$), contraceptive use and intention ($x_4$), exposure to family planning messages on radio in the last few months ($x_5$), exposure to family planning messages on television in the last few months ($x_6$), and exposure to family planning messages in newspapers or magazines in the last few months ($x_7$).

For all three models, the general form of the logistic regression equation was

\[\ln\left(\frac{p}{1-p}\right) = \alpha_0 + \sum_{i=1}^{k} \alpha_i x_i\]

where $p$ denotes the probability of having ever undergone cancer screening, $\ln\left(\frac{p}{1-p}\right)$ represents the log odds (logit) of the outcome, and $\alpha_0, \alpha_1, \ldots, \alpha_k$ are the logistic regression coefficients corresponding to the predictor variables included in each model.

## Results

Table 1 presents the distribution of cancer screening uptake across states and union territories and at the national level, based on the absolute number (N) of women interviewed. At the national level, among the 225,565 women surveyed, only 1.2% reported having ever undergone cervical cancer screening. Breast cancer screening was reported by 0.5% of respondents, while 1,421 women (0.6%) reported screening for oral cancer. Overall, cancer screening coverage in India remains markedly low. Cervical cancer screening was the most commonly reported modality, followed by oral cancer screening, whereas breast cancer screening was the least utilized.

**Table 1:** Distribution of cancer screening uptake across states and union territories and India N: total women interviewed, n: frequency of women reporting screening (“yes”), %: proportion of affirmative responses, NCT: National Capital Territory

State	Total number of women interviewed (N)	Ever screened for cervical cancer, n (%)	Ever screened for breast cancer, n (%)	Ever screened for oral cancer, n (%)
Jammu and Kashmir	1,620	6 (0.4%)	4 (0.2%)	11 (0.7%)
Himachal Pradesh	951	5 (0.5%)	4 (0.4%)	6 (0.6%)
Punjab	3,906	149 (3.8%)	21 (0.5%)	20 (0.5%)
Chandigarh	136	5 (3.7%)	0 (0.0%)	0 (0.0%)
Uttarakhand	1,753	4 (0.2%)	3 (0.2%)	7 (0.4%)
Haryana	4,300	16 (0.4%)	13 (0.3%)	12 (0.3%)
NCT of Delhi	2,822	6 (0.2%)	3 (0.1%)	13 (0.5%)
Rajasthan	14,730	36 (0.2%)	22 (0.1%)	24 (0.2%)
Uttar Pradesh	44,190	403 (0.9%)	122 (0.3%)	225 (0.5%)
Bihar	29,918	113 (0.4%)	89 (0.3%)	124 (0.4%)
Sikkim	58	1 (1.7%)	0 (0.0%)	0 (0.0%)
Arunachal Pradesh	171	1 (0.6%)	1 (0.6%)	1 (0.6%)
Nagaland	213	0 (0.0%)	0 (0.0%)	1 (0.5%)
Manipur	448	5 (1.1%)	5 (1.1%)	3 (0.7%)
Mizoram	176	7 (4.0%)	2 (1.1%)	1 (0.6%)
Tripura	598	2 (0.3%)	1 (0.2%)	3 (0.5%)
Meghalaya	856	7 (0.8%)	3 (0.4%)	2 (0.2%)
Assam	5,791	11 (0.2%)	10 (0.2%)	12 (0.2%)
West Bengal	15,542	14 (0.090%)	15 (0.096%)	12 (0.077%)
Jharkhand	6,847	30 (0.4%)	10 (0.1%)	14 (0.2%)
Odisha	7,112	48 (0.7%)	8 (0.1%)	10 (0.1%)
Chhattisgarh	4,991	20 (0.4%)	7 (0.1%)	7 (0.1%)
Madhya Pradesh	13,424	151 (1.1%)	117 (0.9%)	147 (1.1%)
Gujarat	9,610	24 (0.2%)	8 (0.1%)	16 (0.2%)
Dadra and Nagar Haveli and Daman and Diu	87	0 (0.0%)	0 (0.0%)	0 (0.0%)
Maharashtra	17,905	405 (2.3%)	276 (1.5%)	336 (1.9%)
Andhra Pradesh	7,210	219 (3.0%)	34 (0.5%)	220 (3.1%)
Karnataka	9,909	43 (0.4%)	14 (0.1%)	20 (0.2%)
Goa	200	2 (1.0%)	2 (1.0%)	3 (1.5%)
Lakshadweep	10	0 (0.0%)	0 (0.0%)	0 (0.0%)
Kerala	4,412	59 (1.3%)	46 (1.0%)	13 (0.3%)
Tamil Nadu	10,603	792 (7.5%)	375 (3.5%)	95 (0.9%)
Puducherry	141	8 (5.7%)	4 (2.9%)	1 (0.7%)
Andaman and Nicobar Islands	39	1 (2.6%)	1 (2.6%)	4 (10.3%)
Telangana	4,861	49 (1.01%)	10 (0.2%)	58 (1.2%)
Ladakh	27	0 (0.0%)	0 (0.0%)	0 (0.0%)
India	225,565	2,642 (1.2%)	1,230 (0.5%)	1,421 (0.6%)

States with higher screening uptake

Tamil Nadu reported the highest screening coverage across all three cancers, with 7.5% for cervical cancer, 3.5% for breast cancer, and 0.9% for oral cancer screening. The Andaman and Nicobar Islands demonstrated an exceptionally high oral cancer screening rate of 10.26% (n = 4), substantially exceeding the national average. Maharashtra also showed relatively higher participation, with screening rates of 2.3% (n = 405) for cervical cancer, 1.5% (n = 276) for breast cancer, and 1.9% (n = 336) for oral cancer. Andhra Pradesh reported above-average uptake, particularly for cervical (3.0%) and oral cancer (3.1%) screening. Puducherry similarly demonstrated elevated screening levels, with 5.7% (n = 8) reporting cervical cancer screening and 2.9% (n = 4) reporting breast cancer screening.

States with lower screening uptake

Several states and UTs, including Dadra and Nagar Haveli and Daman and Diu, Ladakh, Lakshadweep, Assam, and Nagaland, reported either zero or negligible participation across all three cancer screening modalities. In West Bengal, among 15,542 women interviewed, only 14 (0.09%) reported cervical cancer screening, 15 (0.10%) reported breast cancer screening, and 12 (0.08%) reported oral cancer screening, indicating extremely low coverage.

Regional patterns

Distinct regional disparities in cancer screening uptake were observed. Northern and central Indian states, such as Uttar Pradesh, Rajasthan, Bihar, Delhi, Haryana, and Madhya Pradesh, exhibited substantial variability but generally low to moderate screening coverage. Despite their large populations, Rajasthan and Uttar Pradesh demonstrated particularly poor participation. In Uttar Pradesh, cervical cancer screening was reported by 0.9% of women, breast cancer screening by 0.3%, and oral cancer screening by 0.5%.

In contrast, southern and western states, including Tamil Nadu, Kerala, Maharashtra, Andhra Pradesh, and Goa, demonstrated relatively higher screening uptake, with Tamil Nadu, Kerala, and Maharashtra showing the most consistent coverage across cancer types. Among the northeastern states, Mizoram (4.0%), Arunachal Pradesh (0.6%), and Manipur (1.1%) reported comparatively higher cervical cancer screening rates. However, Nagaland and Sikkim reported no participation in breast or oral cancer screening (0%), highlighting potential gaps in access to and utilization of preventive services. Overall, cancer screening coverage was highest in the southern and western regions of India, while the northern, central, and northeastern regions continued to lag behind.

Table 2 presents the sociodemographic, reproductive, and ancillary correlates of ever undergoing cervical, breast, and oral cancer screening. The majority of variables demonstrated statistically significant associations with all three screening outcomes, as determined by the chi-square test (p < 0.05).

**Table 2:** Associates of women who have ever undergone screening tests for cervical, breast, and oral cancers χ²: chi-square test used to assess associations between predictor variables and cancer screening status, p < 0.05: statistically significant, n: absolute number of women who reported ever undergoing cervical cancer screening, %: corresponding proportion within each category

Associates	Ever undergone screening for cervical cancer n (%)	Ever undergone breast cancer screening n (%)	Ever undergone a screening for oral cancer n (%)	Total
Sociodemographic determinants				
Place of residence				
Urban	947 (1.6)	518 (0.9)	541 (0.9)	59,204
Rural	1,696 (1.0)	712 (0.4)	880 (0.5)	166,363
Total	2,643 (1.2)	1,230 (0.5)	1,421 (0.6)	225,567
Age				
<20	51 (0.8)	17 (0.3)	22 (0.4)	6,001
20-34	2,321 (1.2)	1,099 (0.5)	1,283 (0.6)	200,970
35-49	271 (1.5)	114 (0.6)	116 (0.6)	18,596
Total	2,643 (1.2)	1,230 (0.5)	1,421 (0.6)	225,567
Highest education level				
No education	260 (0.5)	125 (0.3)	203 (0.4)	48,303
Primary	239 (0.9)	77 (0.3)	137 (0.5)	27,883
Secondary	1,390 (1.2)	598 (0.5)	765 (0.7)	114,621
Higher	754 (2.2)	430 (1.2)	315 (0.9)	34,760
Total	2,643 (1.2)	1,230 (0.5)	1,420 (0.6)	225,567
Caste				
Schedule caste	708 (1.3)	267 (0.5)	298 (0.6)	52,718
Schedule tribe	139 (0.6)	64 (0.3)	98 (0.4)	22,790
Other backward class	1,339 (1.4)	646 (0.7)	650 (0.7)	98,088
None of them	350 (0.9)	164 (0.4)	287 (0.7)	39,792
Don't know	6 (0.3)	8 (0.4)	2 (0.1)	2,087
Total	2,542 (1.2)	1,149 (0.5)	1,335 (0.6)	215,475
Religion				
Hindu	2,133 (1.2)	1,001 (0.6)	1,143 (0.6)	179,506
Muslim	232 (0.6)	123 (0.3)	187 (0.5)	36,278
Christian	132 (2.8)	60 (1.3)	42 (0.9)	4,701
Sikh	108 (3.9)	24 (0.9)	25 (0.9)	2,769
Buddhist/neo-Buddhist	21 (1.9)	16 (1.5)	17 (1.6)	1,085
Jain	14 (4.7)	4 (1.3)	4 (1.3)	300
Others (Jewish, Parsi, no religion, and other)	2 (0.2)	2 (0.2)	2 (0.2)	927
Total	2,642 (1.2)	1,230 (0.5)	1,420 (0.6)	225,566
Wealth index				
Poorest	345 (0.6)	164 (0.3)	266 (0.5)	55,841
Poorer	415 (0.8)	183 (0.4)	225 (0.5)	49,270
Middle	575 (1.3)	266 (0.6)	275 (0.6)	44,277
Richer	661 (1.6)	274 (0.4)	315 (0.8)	41,449
Richest	646 (1.9)	343 (1.0)	341 (1.0)	34,729
Total	2,642 (1.2)	1,230 (0.5)	1,422 (0.6)	225,566
Reproductive health determinants				
Birth order				
1st	1,134 (1.3)	541 (0.6)	613 (0.7)	88,107
2nd and 3rd	1,290 (1.2)	582 (0.5)	677 (0.6)	110,383
4th and 5th	180 (0.8)	89 (0.4)	110 (0.5)	21,738
6th +	39 (0.7)	18 (0.3)	21 (0.4)	5,340
Total	2,643 (1.2)	1,230 (0.5)	1,421 (0.6)	225,568
Currently breastfeeding				
No	1,229 (1.5)	597 (0.7)	560 (0.7)	80,993
Yes	1,414 (1.0)	633 (0.4)	861 (0.6)	144,575
Total	2,643 (1.2)	1,230 (0.5)	1,421 (0.6)	225,568
Ever had a terminated pregnancy				
No	2,186 (1.2)	1,018 (0.5)	1,221 (0.6)	190,017
Yes	457 (1.3)	212 (0.6)	200 (0.6)	35,550
Total	2,643 (1.2)	1,230 (0.5)	1,421 (0.6)	225,567
Current marital status				
Never in union	0 (0.0)	0 (0.0)	1 (0.5)	221
Married	2,621 (1.2)	1,213 (0.5)	1,410 (0.6)	222,983
Widowed	6 (0.5)	4 (0.3)	1 (0.1)	1,276
Divorced	0 (0.0)	0 (0.0)	1 (0.5)	191
No longer living together/separated	15 (1.7)	12 (1.3)	9 (1.0)	895
Total	2,642 (1.2)	1,229 (0.5)	1,422 (0.6)	225,566
Currently residing with husband/partner				
Living with her	2,335 (1.2)	1,079 (0.6)	1,282 (0.7)	194,584
Staying elsewhere	286 (1.0)	134 (0.5)	128 (0.5)	286
Total	2,621 (1.2)	1,213 (0.5 )	1,410 (0.6)	222,982
Contraceptive use and intention				
Using modern methods	1,329 (1.3)	550 (0.5)	626 (0.6)	103,317
Using traditional methods	180 (0.6)	78 (0.3)	86 (0.3)	292,08
Non-user-intends to use later	254 (1.5)	141 (0.8)	156 (0.9)	17,380
Does not intend to use	880 (1.2)	462 (0.6)	553 (0.7)	75,615
Never had sex	0 (0.0)	0 (0.0)	0 (0.0)	46
Total	2,643 (1.2)	1,231 (0.5)	1,421 (0.6)	225,566
Ancillary factors				
Working status				
No	362 (1.3)	145 (0.5)	142 (0.5)	28,499
Yes	80 (1.4)	34 (0.6)	28 (0.5)	5,851
Total	442 (1.2)	179 (0.5)	170 (0.5)	34,350
Body mass index				
Underweight	387 (0.9)	166 (0.4)	210 (0.5)	43,313
Healthy weight	1,403 (1.0)	644 (0.5)	722 (0.5)	137,025
Overweight	588 (1.8)	276 (0.8)	250 (0.8)	32,587
Obesity	235 (2.4)	119 (1.2)	102 (1.0)	9,933
Total	441 (0.3)	180 (0.5)	170 (0.5)	34,316
Husband/partner education				
No education	33 (0.6)	10 (0.2)	17 (0.3)	5,227
Primary	65 (1.4)	13 (0.3)	20 (0.4)	4,570
Secondary	222 (1.2)	100 (0.5)	91 (0.5)	18,730
Higher	121 (2.1)	57 (1.0)	42 (0.7)	5,639
Don't know	0 (0.0)	0 (0.0)	0 (0.0)	150
Total	2,613 (1.2)	1,205 (0.5)	1,284 (0.6)	222,858
Heard family planning on the radio for the last few months				
No	2,100 (1.1)	879 (0.5)	1,081 (0.6)	191,735
Yes	542 (1.6)	351 (1.0)	340 (1.0)	33,831
Total	2,642 (1.2)	1,230 (0.5)	1,421 (0.6)	225,566
Heard family planning on TV for the last few months				
No	862 (0.6)	403 (0.4)	554 (0.6)	98,303
Yes	1,781 (1.4)	827 (0.6)	866 (0.7)	127,265
Total	2,643 (1.2)	1,230 (0.5)	1,420 (0.6)	225,568
Heard about family planning in the newspaper/magazine for the last few months				
No	1,557 (1.0)	701 (0.5)	850 (0.6)	149,413
Yes	1,086 (1.4)	529 (0.7)	571 (0.7)	76,154
Total	2,643 (1.2)	1,230 (0.5)	1,421 (0.6)	225,567

Screening uptake was significantly higher among urban women compared with rural counterparts (cervical: 1.6% vs. 1.0%; breast: 0.9% vs. 0.4%; oral: 0.9% vs. 0.5%; p = 0.00-0.032). Women aged 35-49 years reported the highest cervical cancer screening (1.5%), whereas women under 20 years had the lowest uptake across all cancer types. A clear educational gradient was observed: screening was highest among women with higher education (cervical: 2.2%; breast: 1.2%; oral: 0.9%) and lowest among women with no formal education (≤0.5% across screenings). Socio-cultural disparities were also evident: cervical cancer screening ranged from 0.6% among scheduled tribe women to 1.4% among other backward classes (p = 0.00), and from 0.6% among Muslim women to 4.7% among Jain women. A pronounced wealth gradient was observed (p = 0.00), with the richest women reporting higher uptake (cervical: 1.9%; breast: 1.0%; oral: 1.0%) than the poorest women (≤0.6% across screenings).

Regarding reproductive factors, screening was significantly higher among women with first-order births (breast: 0.6%; oral: 0.7%; p < 0.01), among women not currently breastfeeding (cervical: 1.5% vs. 1.0%; p = 0.00), and among women using or intending to use modern contraceptive methods (p = 0.00). A history of terminated pregnancy was associated with slightly higher cervical cancer screening (1.3%, n = 457; p = 0.03). Married women and those co-residing with their husband or partner also demonstrated higher screening uptake (p < 0.05).

Among ancillary factors, employment status was not significantly associated with screening (p > 0.05). In contrast, BMI showed a positive association, with cervical cancer screening highest among obese women (2.4%). The partner’s education was also influential, with cervical screening rates reaching 2.1% among women whose partners had higher education (p < 0.05).

Table 3 presents the multivariable logistic regression results examining predictors of cervical, breast, and oral cancer screening across three sequential models. Several sociodemographic, reproductive, and ancillary factors remained independently associated with screening uptake after adjustment.

**Table 3:** Multivariable logistic regression analysis of predictors of cancer screening among women in India OR (p) (95% CI): adjusted OR with category-specific p-value and 95% CI, p: overall statistical significance of each predictor variable across all its categories OR: odds ratio, CI: confidence interval, ref: reference category

Predictors	Ever undergone a screening for cervical cancer OR (p) (95% CI)	Ever undergone breast cancer screening OR (p) (95% CI)	Ever undergone a screening for oral cancer OR (p) (95% CI)
Model 1: sociodemographic determinants			
Place of residence	p = 0.00	p = 0.00	p = 0.00
Urban	Ref	Ref	Ref
Rural	0.87 (0.004) (0.79-0.96)	0.68 (0.00) (0.59-0.78)	0.74 (0.004) (0.65-0.84)
Age	p = 0.00	p = 0.02	p = 0.28
Less than 20	Ref	Ref	Ref
20-34	1.13 (0.40) (0.85-1.52)	1.33 (0.25) (0.82-2.15)	1.40 (0.12) (0.92-2.15)
35-49	1.59 (0.04) (1.16-2.18)	1.72 (0.04) (1.02-2.87)	1.45 (0.12) (0.91-2.31)
Highest education level	p = 0.00	p = 0.00	p = 0.001
No education	Ref	Ref	Ref
Primary	1.52 (0.00) (1.27-1.81)	1.03 (0.83) (0.77-1.38)	1.13 (0.28) (0.94-1.41)
Secondary	1.83 (0.00) (1.58-2.11)	1.67 (0.00) (1.35-2.05)	1.35 (0.00) (1.14-1.61)
Higher	2.86 (0.00) (2.43-3.37)	3.48 (0.00) (2.75-4.39)	1.49 (0.00) (1.21-1.85)
Caste	p = 0.00	p = 0.00	p = 0.00
Schedule caste	Ref	Ref	Ref
Schedule tribe	0.52 (0.00) (0.43-0.63)	0.64 (0.002) (0.48-0.84)	0.85 (0.17) (0.67-1.07)
Other backward class	1.02 (0.66) (0.93-1.13)	1.28 (0.001) (1.09-1.48)	1.21 (0.008) (1.05-1.40)
None of them	0.55 (0.00) (0.48-0.63)	0.67 (0.00) (0.55-0.82)	1.23 (0.02) (1.03-1.46)
Religion	p = 0.00	p = 0.00	p = 0.00
Hindu	Ref	Ref	Ref
Muslim	0.58 (0.00) (0.49-0.68)	0.54 (0.00) (0.43-0.68)	0.66 (0.00) (0.55-0.79)
Christian	2.30 (0.00) (1.90-2.79)	1.97 (0.00) (1.47-2.64)	1.17 (0.38) (0.82-1.67)
Sikh	2.88 (0.00) (2.33-3.56)	1.25 (0.37) (0.77-2.00)	0.99 (0.96) (0.62-1.58)
Buddhist/neo-Buddhist	1.36 (0.17) (0.88-2.11)	2.51 (0.00) (1.49-4.21)	2.70 (0.00) (1.66-4.40)
Jain	0.99 (0.98) (0.35-2.77)	1.76 (0.29) (0.62-4.94)	1.51 (0.44) (0.54-4.23)
Others (Jewish, Parsi, no religion, and other)	0.38 (0.19) (0.08-1.64)	0.94 (0.93) (0.25-3.61)	0.58 (0.41) (0.15-2.18)
Wealth index	p = 0.00	p = 0.06	p = 0.09
Poorest	Ref	Ref	Ref
Poorer	1.15 (0.06) (0.99-1.34)	1.03 (0.79) (0.83-1.28)	0.83 (0.05) (0.69-1.00)
Middle	1.51 (0.00) (1.30-1.74)	1.30 (0.02) (1.05-1.61)	0.96 (0.66) (0.78-1.15)
Richer	1.59 (0.00) (1.38-1.86)	1.08 (0.49) (0.86-1.36)	1.03 (0.74) (0.85-1.26)
Richest	1.49 (0.00) (1.27-1.78)	1.15 (0.26) (0.89-1.48)	1.09 (0.43) (0.88-1.36)
Model-2: reproductive health determinants			
Birth order	p = 0.00	p = 0.00	p = 0.001
1st order	Ref	Ref	Ref
2nd and 3rd order	0.88 (0.00) (0.80-0.95)	0.82 (0.00) (0.73-0.93)	0.88 (0.02) (0.79-0.98)
4th and 5th order	0.63 (0.00) (0.54-0.74)	0.66 (0.00) (0.53-0.82)	0.74 (0.00) (0.60-0.90)
6+ order	0.55 (0.00) (0.40-0.76)	0.52 (0.00) (0.32-0.84)	0.56 (0.00) (0.36-0.86)
Currently breastfeeding	p = 0.00	p = 0.00	p = 0.002
No	Ref	Ref	Ref
Yes	0.63 (0.00) (0.59-0.69)	0.59 (0.00) (0.53-0.66)	0.84 (0.00) (0.76-0.94)
Ever had a terminated pregnancy	p = 0.052	p = 0.22	p = 0.100
No	Ref	Ref	Ref
Yes	1.11 (0.05) (0.99-1.23)	1.09 (0.22) (0.94-1.27)	0.88 (0.10) (0.76-1.03)
Currently residing with husband/partner	p = 0.016	p = 0.15	p = 0.15
Living with her	Ref	Ref	Ref
Staying elsewhere	0.86 (0.01) (0.76-0.97)	0.88 (0.15) (0.73-1.05)	0.69 (0.00) (0.57-0.83)
Model 3: ancillary factors			
Working status	p = 0.529	p = 0.46	p = 0.969
No	Ref	Ref	Ref
Yes	1.08 (0.52) (0.85-1.38)	1.15 (0.46) (0.79-1.68)	1.01 (0.97) (0.67-1.52)
Body mass index	p = 0.00	p = 0.00	p = 0.028
Underweight	Ref	Ref	Ref
Healthy weight	0.92 (0.56) (0.71-1.21)	1.35 (0.24) (0.82-2.23)	1.12 (0.62) (0.71-1.78)
Overweight	1.78 (0.00) (1.32-2.40)	3.24 (0.00) (1.90-5.51)	1.86 (0.02) (1.10-3.15)
Obesity	1.04 (0.87) (0.64-1.69)	2.74 (0.00) (1.39-5.41)	1.78 (0.11) (0.88-3.61)
Contraceptive use and intention	p = 0.015	p = 0.165	p = 0.271
Using modern methods	Ref	Ref	Ref
Using traditional methods	0.59 (0.00) (0.43-0.84)	0.76 (0.27) (0.47-1.24)	1.16 (0.57) (0.70-1.90)
Non-user-intends to use later	0.91 (0.59) (0.64-1.29)	1.21 (0.46) (0.73-1.98)	1.78 (0.02) (1.07-2.98)
Does not intend to use	0.76 (0.01) (0.61-0.94)	0.67 (0.03) (0.46-0.98)	1.24 (0.24) (0.87-1.79)
Never had sex	0.00 (1.00) (0.00-0.00)	0.00 (1.00) (0.00-0.00)	0.00 (1.00) (0.00-0.00)
Heard family planning on the radio for the last few months	p = 0.069	p = 0.00	p = 0.00
No	Ref	Ref	Ref
Yes	1.26 (0.06) (0.98-1.60)	2.35 (0.00) (1.68-3.29)	2.47 (0.00) (1.74-3.51)
Heard family planning on TV for the last few months	p = 0.00	p = 0.00	p = 0.017
No	Ref	Ref	Ref
Yes	1.63 (0.00) (1.29-2.07)	2.09 (0.00) (1.39-3.11)	1.66 (0.01) (1.09-2.51)
Heard about family planning in the newspaper/magazine for the last few months	p = 0.00	p = 0.00	p = 0.326
No	Ref	Ref	Ref
Yes	0.99 (0.95) (0.79-1.24)	0.72 (0.06) (0.51-1.01)	1.20 (0.32) (0.83-1.73)
Husband/partner education	p = 0.00	p = 0.001	p = 0.788
No education	Ref	Ref	Ref
Primary	2.06 (0.00) (1.35-3.14)	1.44 (0.39) (0.63-3.29)	1.22 (0.55) (0.64-2.34)
Secondary	1.53 (0.02) (1.06-2.22)	2.31 (0.01) (1.18-4.51)	1.08 (0.79) (0.63-1.84)
Higher	2.50 (0.00) (1.68-3.74)	3.61 (0.00) (1.78-7.31)	1.35 (0.32) (0.74-2.46)

Model 1 (sociodemographic determinants)

Rural residence was consistently associated with significantly lower odds of screening for all three cancers compared with urban residence (OR range: 0.68-0.87). Women aged 35-49 years had higher odds of cervical and breast cancer screening than those aged <20 years, whereas age was not significantly associated with oral cancer screening. Educational attainment demonstrated a strong positive association with screening uptake, with women having higher education exhibiting substantially increased odds across all three cancer screenings (OR range: 1.49-3.48).

Caste and religion remained significant predictors of screening behavior. Scheduled Tribe women and Muslim women had lower odds of undergoing cancer screening, while Sikh and Christian women showed higher odds of cervical cancer screening. Wealth status was independently associated only with cervical cancer screening, with women in higher wealth quintiles demonstrating increased odds of screening.

Model 2 (reproductive determinants)

Higher birth order was inversely associated with cancer screening uptake. For cervical cancer, the odds of screening declined progressively from second- or third-births (OR: 0.88; 95% CI: 0.80-0.95) to six or more births (OR: 0.55; 95% CI: 0.40-0.76), with similar inverse trends observed for breast and oral cancer screening. Currently, breastfeeding women have significantly lower odds of screening for cervical (OR: 0.63; 95% CI: 0.59-0.69), breast (OR: 0.59; 95% CI: 0.53-0.66), and oral cancer (OR: 0.84; 95% CI: 0.76-0.94).

Model 3 (ancillary factors)

Employment status was not significantly associated with cancer screening uptake. In contrast, BMI was positively associated with screening behavior. Overweight women had higher odds of undergoing cervical (OR: 1.78), breast (OR: 3.24), and oral cancer screening (OR: 1.86), while obesity was strongly associated with breast cancer screening (OR: 2.74).

Compared with modern contraceptive users, women using traditional contraceptive methods had significantly lower odds of cervical cancer screening (OR: 0.59; p < 0.001), with no significant differences observed for breast or oral cancer screening. Women not intending to use contraception had reduced odds of cervical (OR: 0.76; p = 0.01) and breast cancer screening (OR: 0.67; p = 0.03). Conversely, women intending to use contraception in the future showed higher odds of oral cancer screening (OR: 1.78; p = 0.02).

Exposure to family planning messages via radio was associated with higher odds of breast (OR: 2.35; p < 0.001) and oral cancer screening (OR: 2.47; p < 0.001), while the association with cervical cancer screening was borderline significant (OR: 1.26; p = 0.06). Television exposure was consistently associated with increased odds of screening across all three cancer types, including cervical (OR: 1.63; p < 0.001), breast (OR: 2.09; p < 0.001), and oral cancer screening (OR: 1.66; p = 0.01). In contrast, exposure to family planning messages through print media (newspapers or magazines) was not significantly associated with any screening outcome.

The partner’s educational attainment demonstrated a strong, graded association with screening behavior. Compared with women whose husbands or partners had no formal education, higher partner education was significantly associated with increased odds of cervical cancer screening, rising from primary (OR: 2.06; 95% CI: 1.35-3.14; p < 0.001) to secondary (OR: 1.53; 95% CI: 1.06-2.22; p = 0.02) and higher education (OR: 2.50; 95% CI: 1.68-3.74; p < 0.001). For breast cancer screening, significant associations were observed among women whose partners had secondary (OR: 2.31; 95% CI: 1.18-4.51; p = 0.01) and higher education (OR: 3.61; 95% CI: 1.78-7.31; p < 0.001), whereas primary education was not significant. In contrast, oral cancer screening showed no significant association with partner’s education across any category (p = 0.788).

Overall, Model 3 demonstrated the strongest and most consistent associations, underscoring the critical role of health communication exposure and household educational context in shaping cancer screening behavior.

## Discussion

This study reveals perilously low national screening coverage for cervical (approximately three in 250 women), breast (one in 200), and oral (three in 500) cancers. These findings corroborate the findings of Borkotoky et al. (2024) and Gopika et al. (2022), both of whom documented widespread underutilization of early detection services in India [22,23]. Despite India’s high cancer burden and the availability of low-cost screening methods, particularly for oral and breast cancers, screening uptake remains alarmingly low. The extremely poor coverage of breast cancer screening underscores critical gaps in early detection efforts under the NP-NCD. Limited programmatic emphasis, inadequate integration within primary healthcare, and low population-level awareness likely contribute to these gaps. Moreover, disruptions to routine preventive health services during the COVID-19 pandemic, which coincided with NFHS-5 fieldwork, may have further suppressed screening uptake.

Marked regional disparities were also evident. Consistent with the spatial clustering patterns reported by Monica and Mishra (2020), southern and western states, particularly Tamil Nadu and Maharashtra, demonstrated relatively higher screening uptake, while screening in northeastern, northern, and central regions remained largely imperceptible [24]. Moran’s I spatial analysis by Nilima et al. (2022) similarly identified “hot spots” in Tamil Nadu and Maharashtra and “cold spots” in eastern and northeastern states [25]. Kalaiarasi Muthu et al. (2024) reported breast cancer screening coverage of approximately 54% in southern zones compared with only 3-5% in central and eastern India, further validating this regional divide. Chakravarti and Patel emphasized the need for context-specific interventions by highlighting substantial district-level variation in Uttar Pradesh [26,27]. The relatively high oral cancer screening observed in the Andaman and Nicobar Islands may reflect localized public health initiatives, enhanced outreach through primary healthcare services, or improved access in smaller population settings; however, this finding should be interpreted cautiously, given the small sample size.

Among northeastern states, Mizoram emerged as an outlier, with comparatively higher screening uptake. Sen et al. (2022) attributed this to strong community engagement and the advantages of a smaller population, which enhance program visibility and reach [28]. In contrast, Changkun et al. (2022), using NFHS-4 data, reported substantially higher screening estimates (approximately 21% for cervical and 9% for breast cancer), which likely reflect broader and less specific definitions of “screening” compared with the more explicit and categorical questions used in NFHS-5 [29].

Pronounced sociodemographic inequities were evident in screening uptake. Urban residence, higher educational attainment, and greater wealth were consistently associated with increased screening, mirroring findings by Ooi et al. (2024), who reported education (OR: 1.88) and high income (OR: 2.27) as key predictors [30]. Similar urban-rural differentials have been documented in Nepal by Lamichhane et al. (2023) [31]. A systematic review by Islam et al. (2021) further identified limited awareness and low educational attainment as principal barriers to breast and cervical cancer screening across low- and middle-income countries [32].

The age-specific pattern observed in this study, peak cervical cancer screening among women aged 30-49 years, is consistent with findings reported by Yadav et al. (2024) [33]. Lower screening participation among Muslim women and Scheduled Tribes aligns with evidence from decomposition analyses by Nilima et al. (2022) and Garg et al. (2023), underscoring the influence of religion and caste on healthcare utilization [25,34]. Nagar et al. (2023) further highlighted the role of cultural beliefs and stigma as major deterrents to screening participation. Although some studies have reported positive associations between screening uptake and women’s employment status [35,36], the absence of a significant association in our analysis may reflect the fact that employment does not uniformly translate into financial autonomy or decision-making power over healthcare in many Indian households. Formal education and cancer awareness, however, remain robust facilitators of screening uptake, consistent with findings by Sawhney et al. (2023) [37].

Our findings also indicate that exposure to family planning messages via television and radio, used here as proxy indicators of broader health awareness, significantly increases the likelihood of screening uptake. While Srinath et al.’s (2023) “7A” accessibility framework does not explicitly emphasize mass media influences [38], the wider reach and accessibility of television and radio may explain their stronger associations than those of print media, which relies on literacy and has comparatively limited penetration. The positive association between a partner’s education and screening uptake highlights the critical role of household decision-making dynamics, supportive environments, and facilitated access to preventive healthcare. Similarly, associations with contraceptive use and higher BMI may reflect increased contact with healthcare services, thereby enhancing opportunities for screening.

Conversely, lower screening uptake among women with higher parity and those currently breastfeeding suggests that caregiving responsibilities and competing domestic demands constrain access to preventive health services. Consistent with Subramanian et al. (2024), no significant association was observed between screening uptake and either employment status or history of pregnancy termination [39].

This study uniquely mapped cancer screening patterns across all Indian states and key population subgroups using NFHS-5 data, a large, nationally representative sample. Nonetheless, several limitations warrant consideration. The analysis relied on self-reported data, which introduced the potential for misclassification, recall bias, and respondent misunderstanding, particularly because a single survey item captured multiple screening types. Additionally, NFHS-5 includes only women aged 15-49 years, whereas the risk of cervical and breast cancer extends beyond this age range. The cross-sectional design precludes causal inference, and generalizability beyond India is limited due to contextual differences in healthcare systems and sociocultural norms.

Taken together, although “true” screening levels may be underestimated, the findings unequivocally demonstrate extremely low and uneven cancer screening coverage across India. These results underscore the urgent need for strengthened, context-sensitive screening strategies to reduce preventable cancer morbidity and mortality nationwide.

## Conclusions

Cancer screening patterns in India, as revealed by the model-based findings, are shaped by a complex interplay of sociodemographic, reproductive, and ancillary factors. Urban residence, higher educational attainment, and belonging to non-scheduled caste/scheduled tribe groups consistently emerged as the strongest predictors of higher screening uptake. Women aged 35-49 years and those in higher wealth quintiles were also more likely to undergo cervical and breast cancer screening. Notably, Muslim women were significantly less likely to participate in any form of cancer screening, underscoring persistent religious and cultural disparities in access to preventive healthcare services.

Reproductive factors, including higher birth order, current breastfeeding, and not co-residing with a husband or partner, were strong negative predictors of screening across all three cancer types. In contrast, a history of pregnancy termination was not significantly associated with screening behavior. Among ancillary factors, overweight status, exposure to family planning messages, particularly through radio and television, and higher educational attainment of the husband or partner were positively associated with cancer screening uptake. Conversely, use of traditional contraceptive methods or lack of intention to use contraception was associated with lower screening uptake, especially for cervical and breast cancers.

In light of the observed sociodemographic disparities, targeted interventions are essential to improve equity in cancer screening access. Priority strategies include deploying mobile screening units in underserved rural areas, strengthening routine screening integration within primary healthcare services, and sustaining community-based outreach through frontline health workers to engage women with lower educational attainment. Additionally, culturally tailored awareness initiatives addressing caste- and religion-specific barriers, along with strengthened community-level referral linkages, may further enhance screening uptake among disadvantaged populations.

## References

[1] World Health Organization (2024 (2025). Noncommunicable diseases. https://www.who.int/news-room/fact-sheets/detail/noncommunicable-diseases.

[2] (2025). Data visualization tools for exploring the global cancer burden in 2022. https://gco.iarc.who.int/today/en.

[3] Bray F, Laversanne M, Sung H, Ferlay J, Siegel RL, Soerjomataram I, Jemal A (2024). Global cancer statistics 2022: GLOBOCAN estimates of incidence and mortality worldwide for 36 cancers in 185 countries. CA Cancer J Clin.

[4] Filho AM, Laversanne M, Ferlay J (2025). The GLOBOCAN 2022 cancer estimates: data sources, methods, and a snapshot of the cancer burden worldwide. Int J Cancer.

[5] (2025). Noncommunicable diseases country profiles 2018. https://www.who.int/publications/i/item/ncd-country-profiles-2018.

[6] (2025). Noncommunicable diseases progress monitor 2025. https://www.who.int/publications/i/item/9789240105775.

[7] Sathishkumar K, Chaturvedi M, Das P, Stephen S, Mathur P (2022). Cancer incidence estimates for 2022 and projection for 2025: result from National Cancer Registry Programme, India. Indian J Med Res.

[8] Lentz R, Benson AB 3rd, Kircher S (2019). Financial toxicity in cancer care: prevalence, causes, consequences, and reduction strategies. J Surg Oncol.

[9] Mehlis K, Witte J, Surmann B (2020). The patient-level effect of the cost of cancer care - financial burden in German cancer patients. BMC Cancer.

[10] Carrera PM, Kantarjian HM, Blinder VS (2018). The financial burden and distress of patients with cancer: understanding and stepping-up action on the financial toxicity of cancer treatment. CA Cancer J Clin.

[11] Yadav J, Menon GR, John D (2021). Disease-specific out-of-pocket payments, catastrophic health expenditure and impoverishment effects in India: an analysis of national health survey data. Appl Health Econ Health Policy.

[12] Pramesh S, Badwe A, Borthakur B (2014). Delivery of affordable and equitable cancer care in India. Lancet Oncol.

[13] Dwivedi P, Lohiya A, Bahuguna P (2023). Cost-effectiveness of population-based screening for oral cancer in India: an economic modelling study. Lancet Reg Health Southeast Asia.

[14] Karan A, Engelgau M, Mahal A (2010). The Economic Implications of Non-communicable Disease for India (English). https://documents.worldbank.org/en/publication/documents-reports/documentdetail/488911468041673131.

[15] Sankaranarayanan R, Swaminathan R, Brenner H, Chen K, Chia KS, Chen JG (2010). Cancer survival in Africa, Asia, and Central America: a population-based study. Lancet Oncol.

[16] (2025). The India state-level disease burden initiative. New Delhi, India.

[17] (2025). Cervical cancer screening. https://www.who.int/data/gho/indicator-metadata-registry/imr-details/3240.

[18] Sankaranarayanan R, Ramadas K, Thomas G (2005). Effect of screening on oral cancer mortality in Kerala, India: a cluster-randomised controlled trial. Lancet.

[19] (2025). Towards a cancer-free India: commitment to prevention, treatment and innovation. https://www.pib.gov.in/PressReleasePage.aspx?PRID=2102729&reg=3&lang=2.

[20] International Institute for Population Sciences (2022). International Institute for Population Sciences and ICF: National Family Health Survey 5. International Institute for Population Sciences and ICF: National Family Health Survey 5.

[21] Ministry of Health and Family Welfare (2021). International Institute for Population Sciences (IIPS) and ICF: National Family Health Survey 5 (NFHS 5), India report, Vol- II. Government of India, Mumbai, India 2019-2021. National Family Health Survey (NFHS-5) 2019-21.

[22] Borkotoky K, Singh L, Singh PK, Singh S (2024). Learning from the Indian National Family Health Survey to assess population based oral, cervix and breast cancer screening in low-and-middle income countries. Lancet Reg Health Southeast Asia.

[23] Gopika MG, Prabhu PR, Thulaseedharan JV (2022). Status of cancer screening in India: an alarm signal from the National Family Health Survey (NFHS-5). J Family Med Prim Care.

[24] Monica Monica, Mishra R (2020). An epidemiological study of cervical and breast screening in India: district-level analysis. BMC Womens Health.

[25] Nilima N, Mani K, Kaushik S, Rai SN (2022). Cervical cancer screening and associated barriers among women in India: a generalized structural equation modeling approach. Cancers (Basel).

[26] Kailarasi M, Bhuvaneswari N, Valarmathi S, Sundar JS, Kalpana S, Srinivas G (2024). Breast cancer screening among adult women aged 30 to 49 years: insights and analysis of NFHS 5 (2019-2021). World J Adv Res Rev.

[27] Chakravarti P, Patel KK, Budukh A (2024). Cancer screening uptake by women from India's largest state Uttar Pradesh: district-wise analysis from the fifth round of National Family Health Survey (2019-2021). Ecancermedicalscience.

[28] Sen S, Khan PK, Wadasadawala T, Mohanty SK (2022). Socio-economic and regional variation in breast and cervical cancer screening among Indian women of reproductive age: a study from National Family Health Survey, 2019-21. BMC Cancer.

[29] Changkun Z, Bishwajit G, Ji L, Tang S (2022). Sociodemographic correlates of cervix, breast and oral cancer screening among Indian women. PLoS One.

[30] Min Feng Ooi B, Muschialli L, Kondal D (2024). Individual-level determinants of breast and cervical cancer screening and early testing in two regionally representative urban Indian populations. Prev Med Rep.

[31] Lamichhane B, Adhikari B, Poudel L (2024). Factors associated with uptake of breast and cervical cancer screening among Nepalese women: evidence from Nepal Demographic and Health Survey 2022. PLOS Glob Public Health.

[32] Islam RM, Billah B, Hossain MN, Oldroyd J (2017). Barriers to cervical cancer and breast cancer screening uptake in low-income and middle-income countries: a systematic review. Asian Pac J Cancer Prev.

[33] Yadav N, Nilima KS, Kumari N (2024). Factors influencing cervical cancer screening among reproductive age group women in India: a multilevel analysis of nationwide survey 2019-2021. Glob Transit.

[34] Garg P, Krishnamoorthy Y, Halder P (2025). Urban-rural disparities in cervical cancer screening among Indian women between 30-49 years: a geospatial and decomposition analysis using a nationally representative survey. BMC Cancer.

[35] Nagar A, Madamanchi D, Nair GR (2024). Barriers to cancer diagnosis and treatment: a pilot qualitative study of patient and practitioner perspectives in rural India. Cureus.

[36] Bawalle AA, Nguyen TX, Khan MS, Kadoya Y (2024). Impact of financial literacy and education on breast and cervical cancer screening participation in Japan. PLoS One.

[37] Sawhney R, Nathani P, Patil P (2023). Recognising socio-cultural barriers while seeking early detection services for breast cancer: a study from a Universal Health Coverage setting in India. BMC Cancer.

[38] Srinath A, van Merode F, Rao SV, Pavlova M (2023). Barriers to cervical cancer and breast cancer screening uptake in low- and middle-income countries: a systematic review. Health Policy Plan.

[39] Subramanian SS, Bhaskarapillai B, Jayakrishnan R (2024). Female cancer screening in India: results from the National Family Health Survey (NFHS-5) and the way forward. J Family Med Prim Care.

